# Managing Seroma Formation Post Breast Surgery through Somatostatin Analogs

**DOI:** 10.31662/jmaj.2023-0086

**Published:** 2023-06-30

**Authors:** Aiko Nagayama, Yuko Kitagawa

**Affiliations:** 1 Keio University School of Medicine, Department of Surgery, Tokyo, Japan

**Keywords:** Breast surgery, Axillary dissection, Somatostatin, Seroma

After axillary dissection, the lack of sufficient lymphatic drainage of the breast and the axilla can lead to seroma formation, which is an abnormal collection of serous fluid. The reported incidence rate of seroma can vary from 15% to 90% and is known as one of the most common complications of breast cancer surgery ^(1)^. Seroma can be a source of morbidity and discomfort among patients and, if untreated, could lead to delayed wound healing, skin flap necrosis, and infection. It is usually diminished with drain placements in the axillary space or managed via percutaneous aspiration after drains are removed. Multiple risk factors for seroma are suggested, including age, body weight, hypertension, number of lymph nodes removed, and extent of surgery ^(1)^. A few patients with seroma need significant clinical intervention for their prolonged or severe symptoms. The placement of suction drains, use of fibrin glue, restricting the use of arm, and compression dressings are reported as preventive measures for seroma formation; however, the only clinically meaningful and established method is the placement of drains ^(1)^.

Somatostatin is a cyclic polypeptide derived from a precursor protein processed into several peptide hormones such as somatostatin-14, somatostatin-28, and neuronostatin ^(2)^. Octreotide acetate, a somatostatin analog, is the major treatment option for acromegaly and gastroenteropancreatic neuroendocrine tumor, as well as for managing intestinal fistula (Table 1) ^(2)^. The splanchnic blood flow inhibition and triglyceride absorption by somatostatin are believed to reduce lymphatic fluid ^(3)^.

**Table 1.** Approved Somatostatin Analogs in Japan.

Drug	Administration	Mechanism of action	Indications per label
Octreotide	Subcutaneous injection	High affinity for SSTR2 and inhibits the proliferation of the cells expressing *SSTR2* gene by activating the tyrosine phosphatase pathway	Gastrointestinal hormone-producing tumors (VIP-producing tumors, carcinoid tumors with carcinoid syndrome, gastrin-producing tumors) Acromegaly and pituitary gigantism Improvement of gastrointestinal symptoms associated with gastrointestinal obstruction in palliative care for patients with advanced or recurrent cancer Hypoglycemia associated with congenital hyperinsulinemia
Octreotide LAR	Intramuscular injection		Gastrointestinal hormone-producing tumors (VIP-producing tumors, carcinoid tumors with carcinoid syndrome, gastrin-producing tumors) Acromegaly and pituitary gigantism Hypoglycemia associated with congenital hyperinsulinemia
Lanreotide	Subcutaneous injection	A cyclic octapeptide that was developed with the intent to achieve a longer acting analog	Acromegaly and pituitary gigantism Thyroid-stimulating hormone-producing pituitary tumor
Pasireotide LAR	Intramuscular injection	High affinity to SSTR1, SSTR3, and SSTR5 and with the same affinity to SST2 with octreotide	Cushing Disease

LAR: long-acting release, SSTR: somatostatin receptor, VIP: Vasoactive intestinal peptide A list of approved somatostatin analogs in Japan and their mechanism of action has been given. Octreotide, octreotide long-acting release (LAR), lanreotide, and pasireotide LAR are approved for gastrointestinal hormone-producing tumors, acromegaly, and pituitary gigantism and octreotide is approved for the palliation of gastrointestinal symptoms due to obstruction by advanced cancer.

Hirono et al. conducted a systematic review and meta-analysis to evaluate the efficacy and safety of somatostatin analog after axillary lymph node dissection in patients with breast cancer ^(4)^. This study included seven randomized control trials and compared the volume of drained fluid, duration of drainage, seroma incidence, as well as adverse events such as hematoma, infection, and the length of hospital stay. They found a slight reduction in the lymphatic drainage volume but not a significant reduction in the duration and incidence of seroma. Based on the limited efficacy and some adverse event reports such as vomiting, diarrhea, and infection site pain, the authors concluded that somatostatin analogs are not much effective or safe for seroma management after breast surgery. Although seroma formation could be a source of more serious complications such as surgical site infection or slow wound healing in addition to the patients’ social/economic burden due to prolonged hospital stay or frequent visits, the conventional care of percutaneous aspiration would remain as a mainstay for its management.

The analysis of “who is at high risk of prolonged seroma formation” and “who would benefit from adding somatostatin analog treatment to the current standard management of seroma” is still in question. For example, Prajapati et al. assessed the efficacy of octreotide after modified radical mastectomy and demonstrated the statistically significant seroma volume reduction with octreotide usage ^(5)^. However, the phase 3 randomized trial by Gauthier et al. did not show a significant reduction of lymphatic fluid with the lanreotide injection use in patients who underwent lumpectomy/mastectomy with axillary lymph node dissection ^(3)^. In clinical practice, the extent of surgery seems to be one of the important factors determining the risk of severe or prolonged seroma. Additionally, the conflicting results from multiple trials may be a result of patient/surgical background variability. Moreover, the management of drains after surgery may influence the severity or duration of the seroma.

To our knowledge, somatostatin analog treatment is not routinely recommended for patients who undergo axillary lymph node dissection. More reasonably, patients with prolonged seroma or at high risk of significant seroma can be good candidates for more intervention. In the future, well-designed, prospective trials, defining patients at risk for complicated seroma will be needed.

## Article Information

### Conflicts of Interest

Y Kitagawa received lecture fees from ASUKA Pharmaceutical Holdings and scholarship donation from TEIJIN Pharma.

### Disclaimer

Yuko Kitagawa is one of the Editors of JMA Journal and on the journal‘s Editorial Staff. He was not involved in the editorial evaluation or decision to accept this article for publication at all.
